# Author Correction: Stretching muscle cells induces transcriptional and splicing transitions and changes in SR proteins

**DOI:** 10.1038/s42003-022-04064-7

**Published:** 2022-10-10

**Authors:** Emma R. Hinkle, R. Eric Blue, Yi-Hsuan Tsai, Matthew Combs, Jacquelyn Davi, Alisha R. Coffey, Aladin M. Boriek, Joan M. Taylor, Joel S. Parker, Jimena Giudice

**Affiliations:** 1 Department of Cell Biology and Physiology, The University of North Carolina at Chapel Hill, Chapel Hill, 27599 NC USA; 2 Curriculum in Genetics and Molecular Biology (GMB), The University of North Carolina at Chapel Hill, Chapel Hill, 27599 NC USA; 3 Lineberger Comprehensive Cancer Center, The University of North Carolina at Chapel Hill, Chapel Hill, 27599 NC USA; 4 Department of Pathology and Laboratory Medicine, The University of North Carolina at Chapel Hill, Chapel Hill, 27599 NC USA; 5 Department of Medicine, Baylor College of Medicine, Houston, TX USA; 6 McAllister Heart Institute, The University of North Carolina at Chapel Hill, Chapel Hill, 27599 NC USA

**Keywords:** Gene expression, Transcription

Correction to: *Communications Biology* 10.1038/s42003-022-03915-7, published online 19 September 2022.

In the original version of the Article, the Cell stretching paragraph of the Method section incorrectly stated, “The stretching protocol was as follows: ½ sine, 16% equibiaxial stretch, DC = 50%, and FRSQ = 1, 0.5 Hz (1 s stretch, 1 s relaxation)”.

The text should read, “The stretching protocol was as follows: ½ sine, 16% equibiaxial stretch, DC = 50%, and FREQ = 1 Hz”.

The error has been corrected in both the PDF and HTML versions of the Article.

