# Supplementary material for: Stretching muscle cells induces transcriptional and splicing transitions and changes in SR proteins
Source: Commun Biol. 2022 Sep 19;5:987. doi: 10.1038/s42003-022-03915-7 (PMC9485123; doi:10.1038/s42003-022-03915-7)
Supplement: Supplementary file 2 — Supplementary Information [file 42003_2022_3915_MOESM2_ESM.pdf]

Stretching muscle cells induces transcriptional and splicing transitions and changes in SR proteins

Emma R. Hinkle[1,2], R. Eric Blue[1], Yi-Hsuan Tsai, Matthew Combs[3], Jacquelyn Davi[1], Alisha R. Coffey[4], Aladin M. Boriek[5], Joan M. Taylor[3,6], Joel S. Parker[2,4], Jimena Giudice[1,2,6*]

1. Department of Cell Biology and Physiology, The University of North Carolina at Chapel Hill, Chapel Hill, NC 27599, USA. **2.** Curriculum in Genetics and Molecular Biology (GMB), The University of North Carolina at Chapel Hill, Chapel Hill, NC 27599, USA. **3.** Department of Pathology and Laboratory Medicine, The University of North Carolina at Chapel Hill, Chapel Hill, NC 27599, USA. **4.** Lineberger Comprehensive Cancer Center, The University of North Carolina at Chapel Hill, Chapel Hill, NC 27599, USA. **5.** Department of Medicine, Baylor College of Medicine, Houston, TX 77030, USA. **6.** McAllister Heart Institute, The University of North Carolina at Chapel Hill, Chapel Hill, NC 27599, USA.

*****correspondence jimena_giudice@med.unc.edu

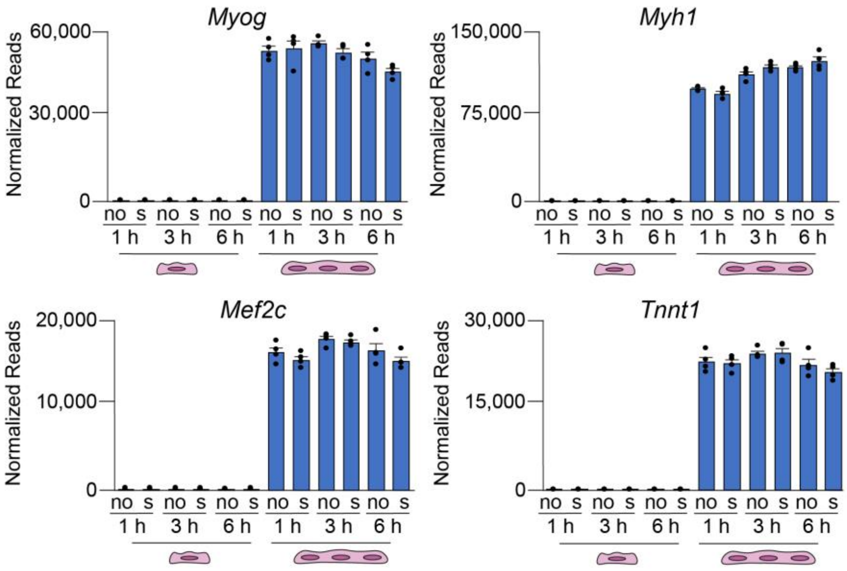

SUPPLEMENTAL FIGURE 1

Supplemental Figure 1. Expression of myogenic markers in myoblasts and differentiated cells. Normalized RNA-seq reads were utilized to compare the mRNA expression levels of the following myogenic markers in myoblasts and differentiated cells: myogenin (*Myog*), myosin heavy chain 1 (*Myh1*), myocyte enhancer factor 2c (*Mef2c*), and troponin T1 (*Tnnt1*). Results are shown as mean ± SEM. no: non-stretched samples. s: stretched samples.

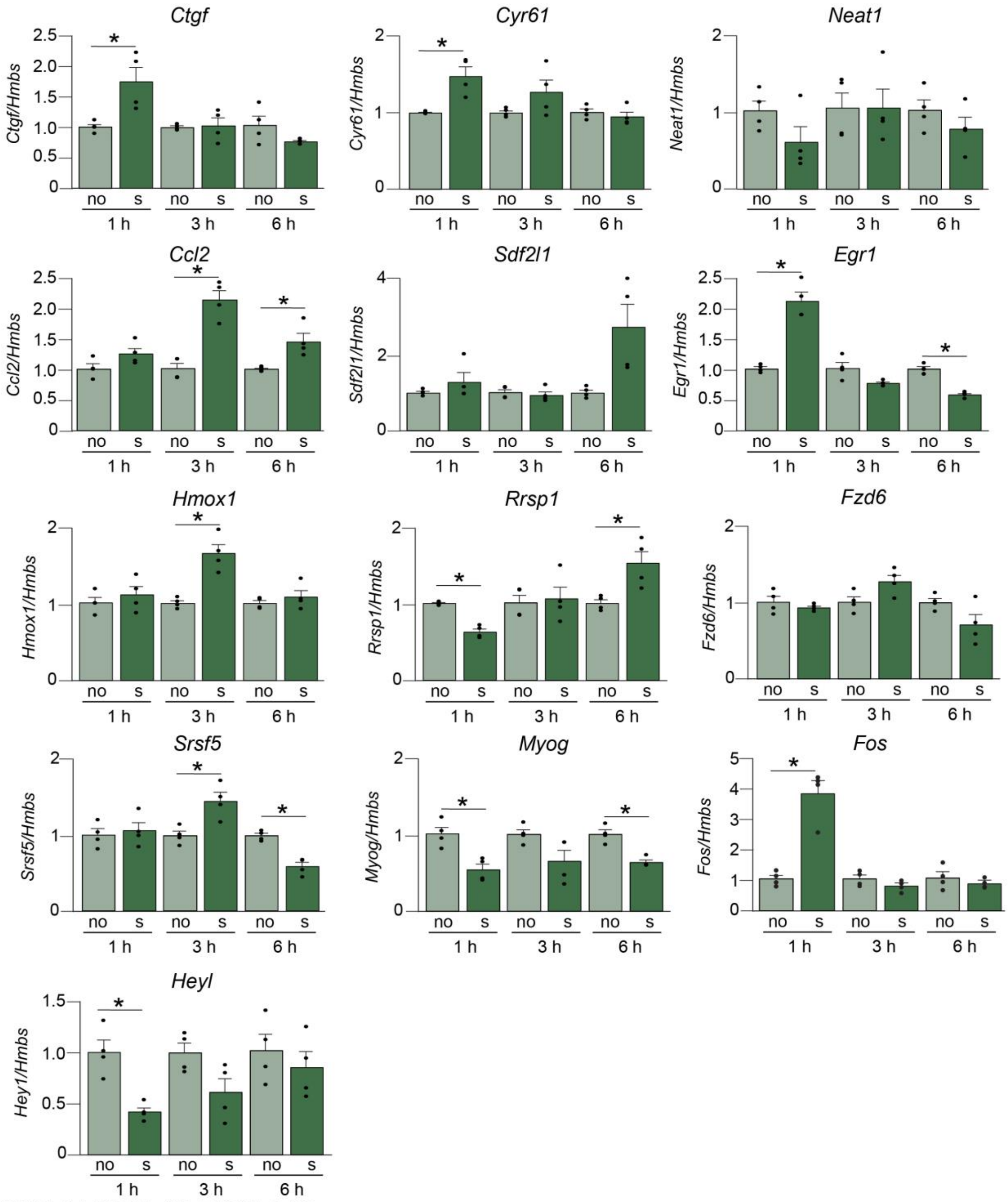

SUPPLEMENTAL FIGURE 2

Supplemental Figure 2. Gene expression changes in response to stretching of myoblasts. Gene expression changes were evaluated by qPCR for 13 genes in myoblasts. The stretched samples were normalized to the non-stretched samples from

the same time point. These experiments were utilized for correlation plots shown in **Figure 1e**. Results are shown as mean ± SEM, *$p < 0.05$ Welch's T-test, $N = 4$. no: non-stretched samples. s: stretched samples.

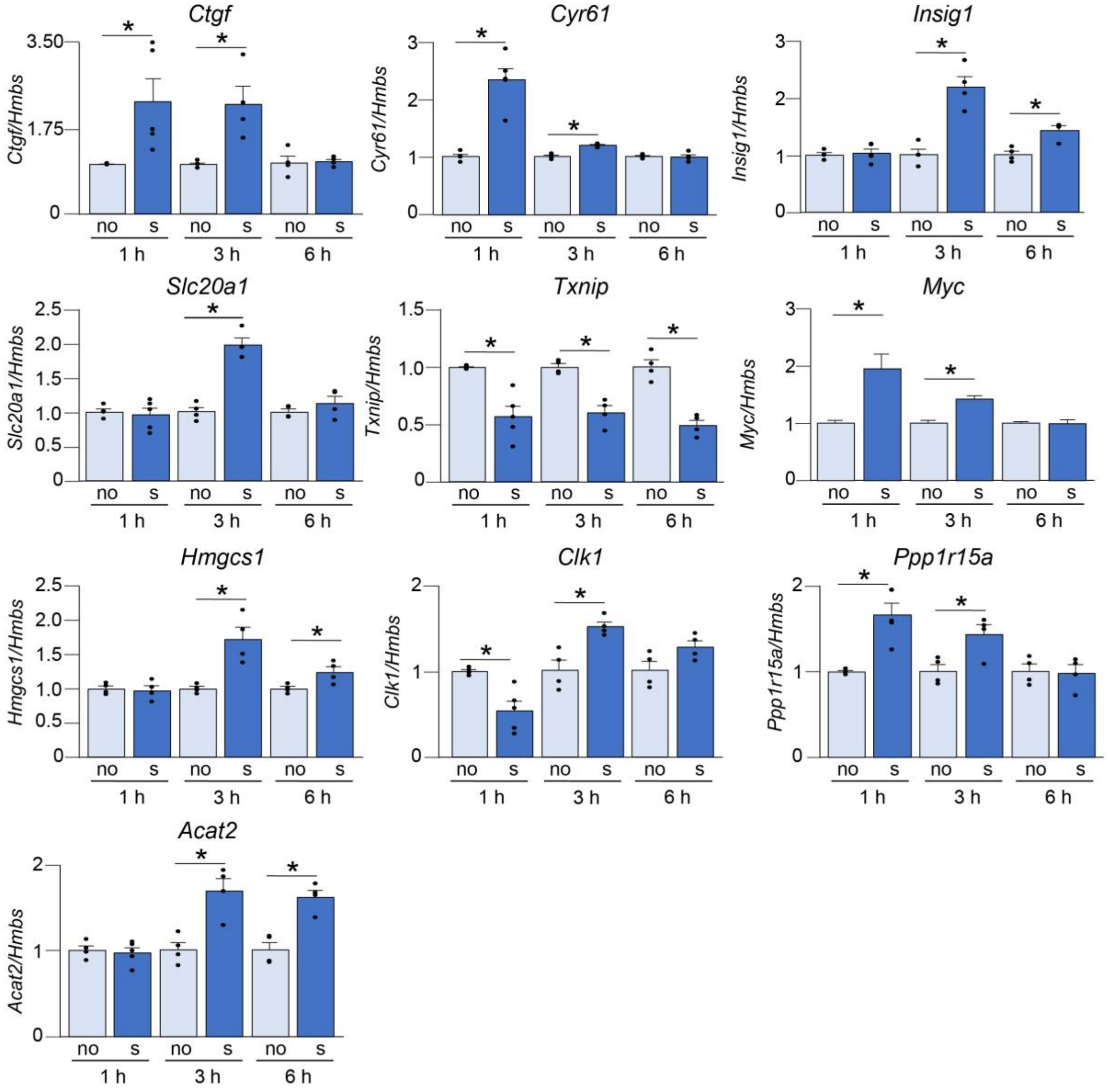

SUPPLEMENTAL FIGURE 3

Supplemental Figure 3. Gene expression changes in response to stretching of differentiated muscle cells. Gene expression changes were evaluated by qPCR for 10 genes in differentiated muscle cells. The stretched samples were normalized to the non-stretched samples from the same time point. These experiments were utilized for

correlation plots shown in **Figure 1f**. Results are shown as mean ± SEM, $*p \leq 0.05$ Welch's T-test, $N = 4\text{-}5$. no: non-stretched samples. s: stretched samples.

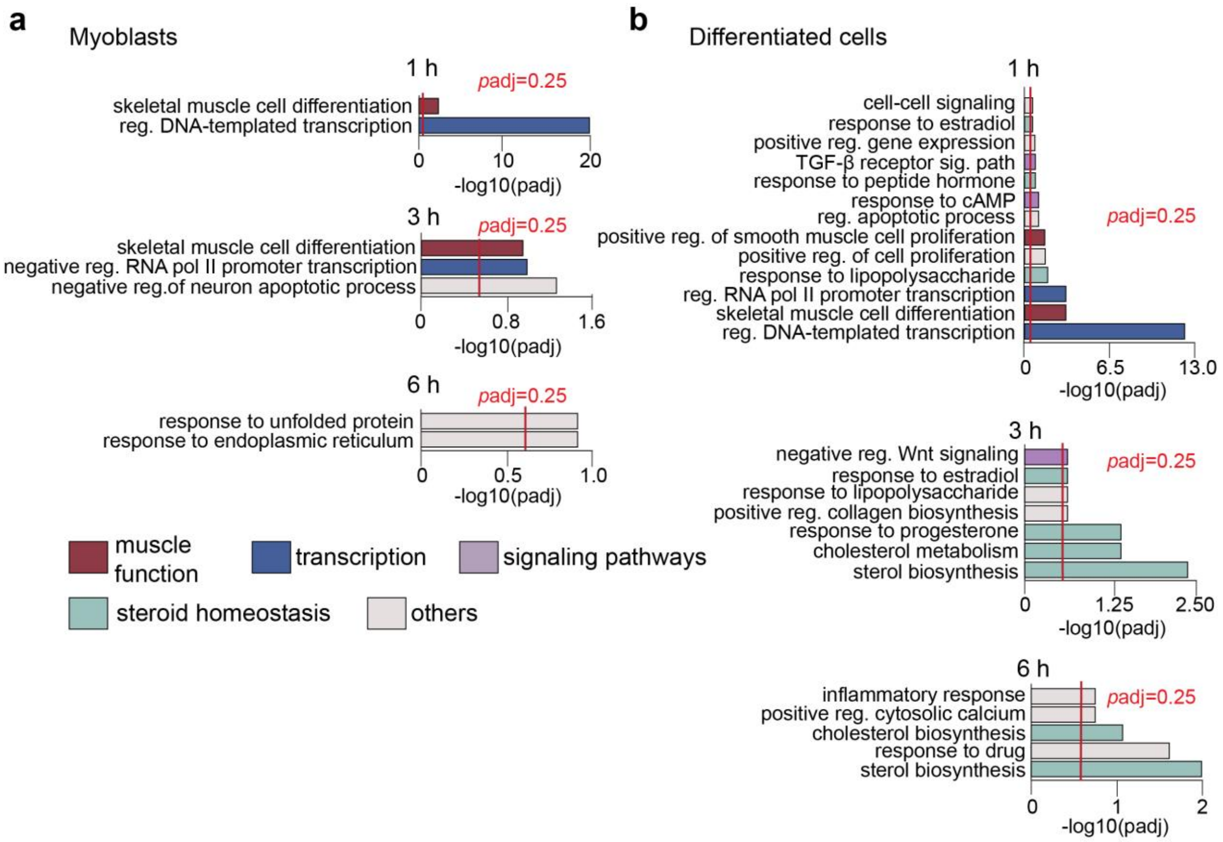

SUPPLEMENTAL FIGURE 4

Supplemental Figure 4. Differentially expressed genes after stretching encode proteins that are involved in muscle development, signaling, steroid metabolism, and protein folding. Gene ontology (GO) analysis of the differentially expressed genes in response to stretching in myoblasts (**a**) and differentiated cells (**b**) was performed using DAVID (Database for Annotation, Visualization, and Integrated Discovery). Red lines indicate adjusted *p*-values (*p*adj, Benjamini-Hochberg) for the GO analysis of 0.25 which is a well-accepted threshold for hypothesis generation for GO[70]. Dev: development. Reg: regulation. RNA Pol II: RNA Polymerase II.

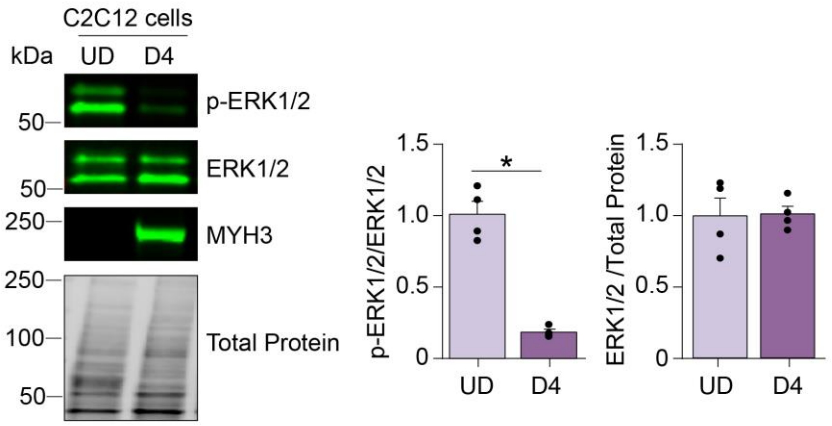

SUPPLEMENTAL FIGURE 5

Supplemental Figure 5. Phosphorylation of ERK1/2 in myoblasts and differentiated cells. Phosphorylation of ERK1/2 (p-ERK1/2) and total levels of ERK1/2 were assayed in undifferentiated myoblasts (UD) and cells differentiated for four days (D4) by western blotting. Myosin heavy chain 3 (MYH3) protein expression was assayed to verify differentiation status of the samples. Results are shown as mean ± SEM, *N*=4. *p<0.05 Welch's T-test.

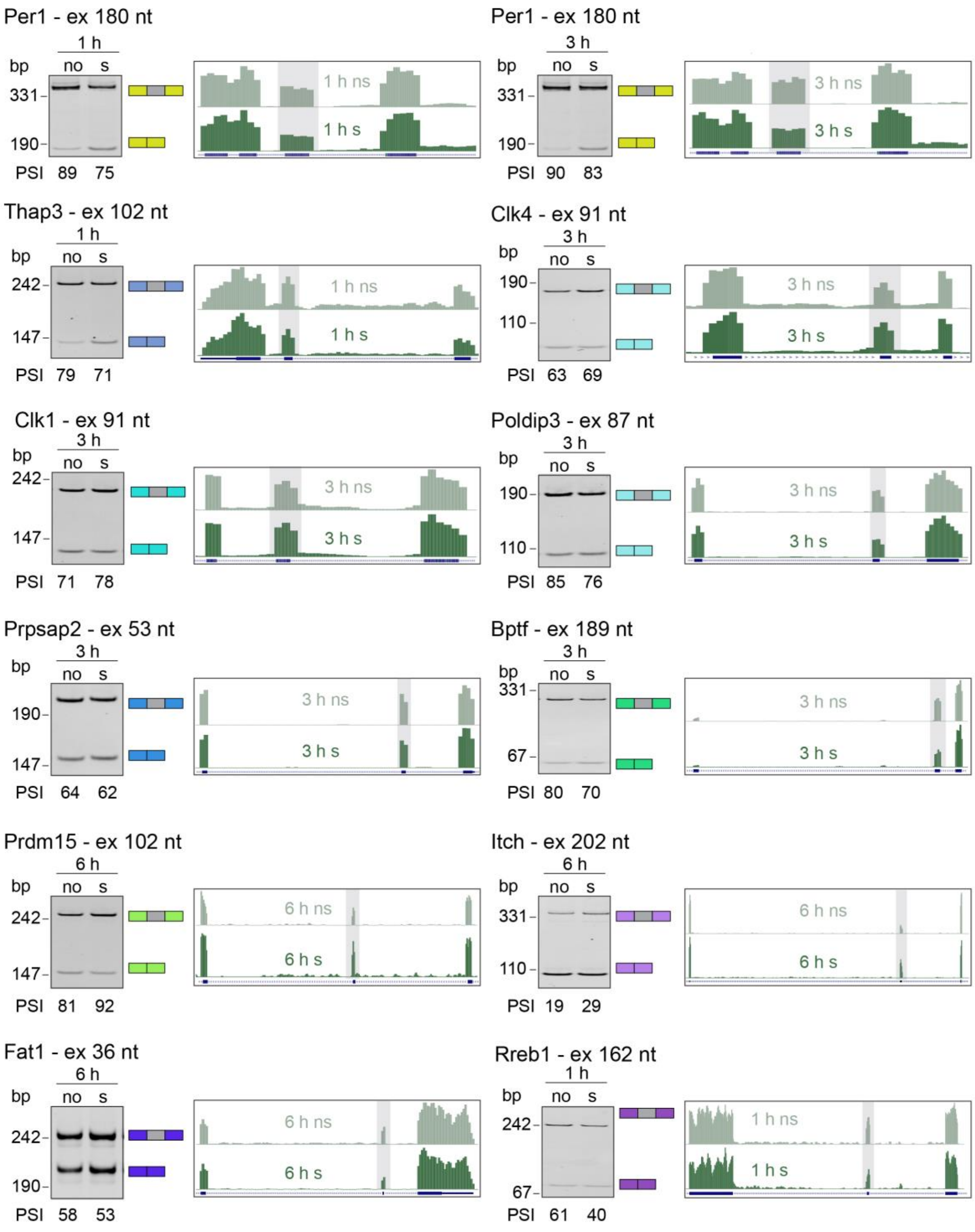

SUPPLEMENTAL FIGURE 6

Supplemental Figure 6. Cassette exon splicing changes in response to stretching of myoblasts. Alternative splicing patterns for 12 events were evaluated by RT-PCR and quantified by densitometry. UCSC browser tracks that correspond to the

gels are shown for each event. These experiments were utilized for correlation plots shown in **Figure 4d**. *N* = 4. PSI: percent spliced in. no: non-stretched samples. s: stretched samples.

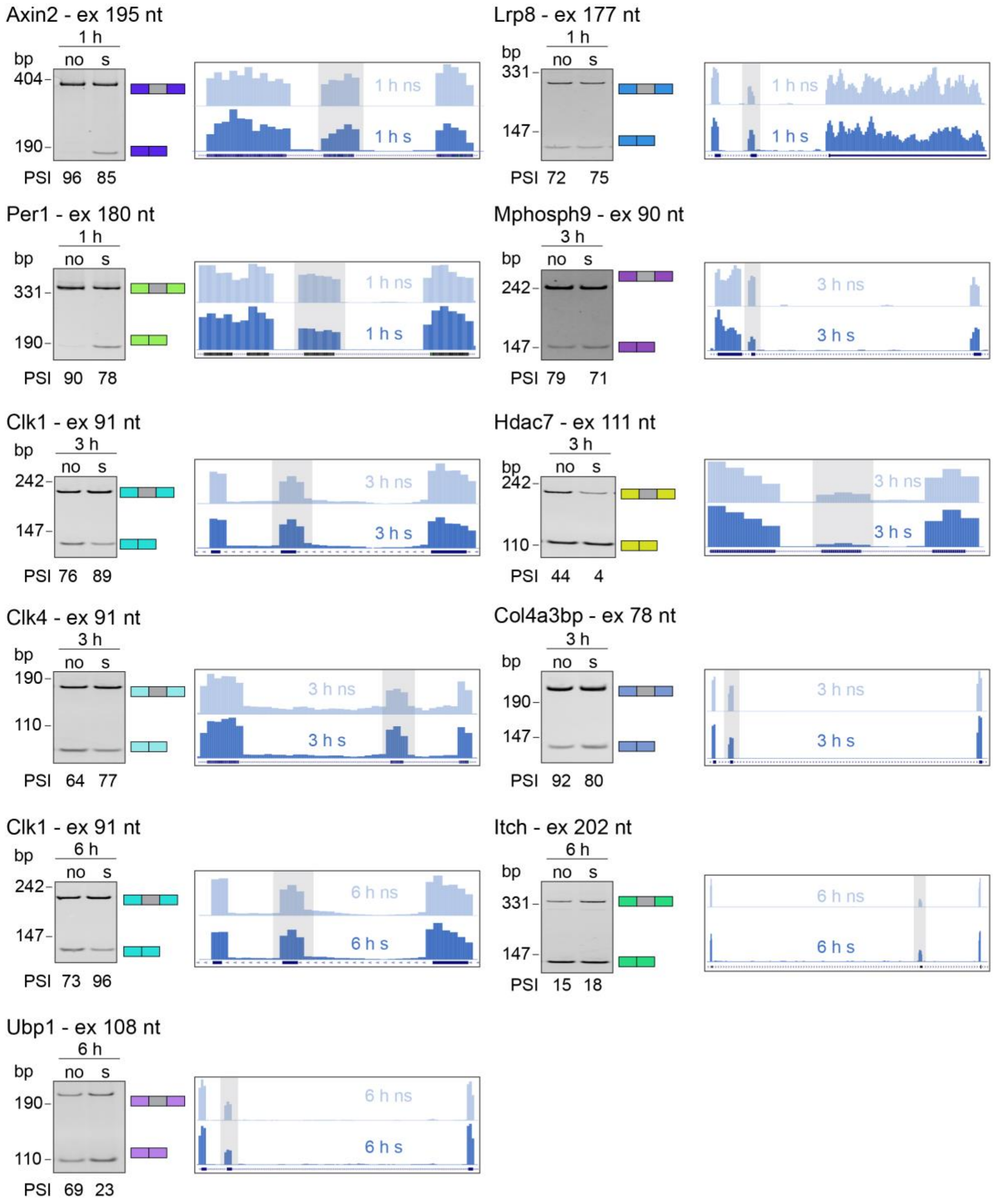

SUPPLEMENTAL FIGURE 7

Supplemental Figure 7. Cassette exon splicing changes in response to stretching of differentiated muscle cells. Alternative splicing patterns for 11 events were evaluated by RT-PCR and quantified by densitometry. UCSC browser tracks that

correspond to the gels are shown for each event. These experiments were utilized for correlation plots shown in **Figure 4e**. N = 5. PSI: percent spliced in. no: non-stretched samples. s: stretched samples.

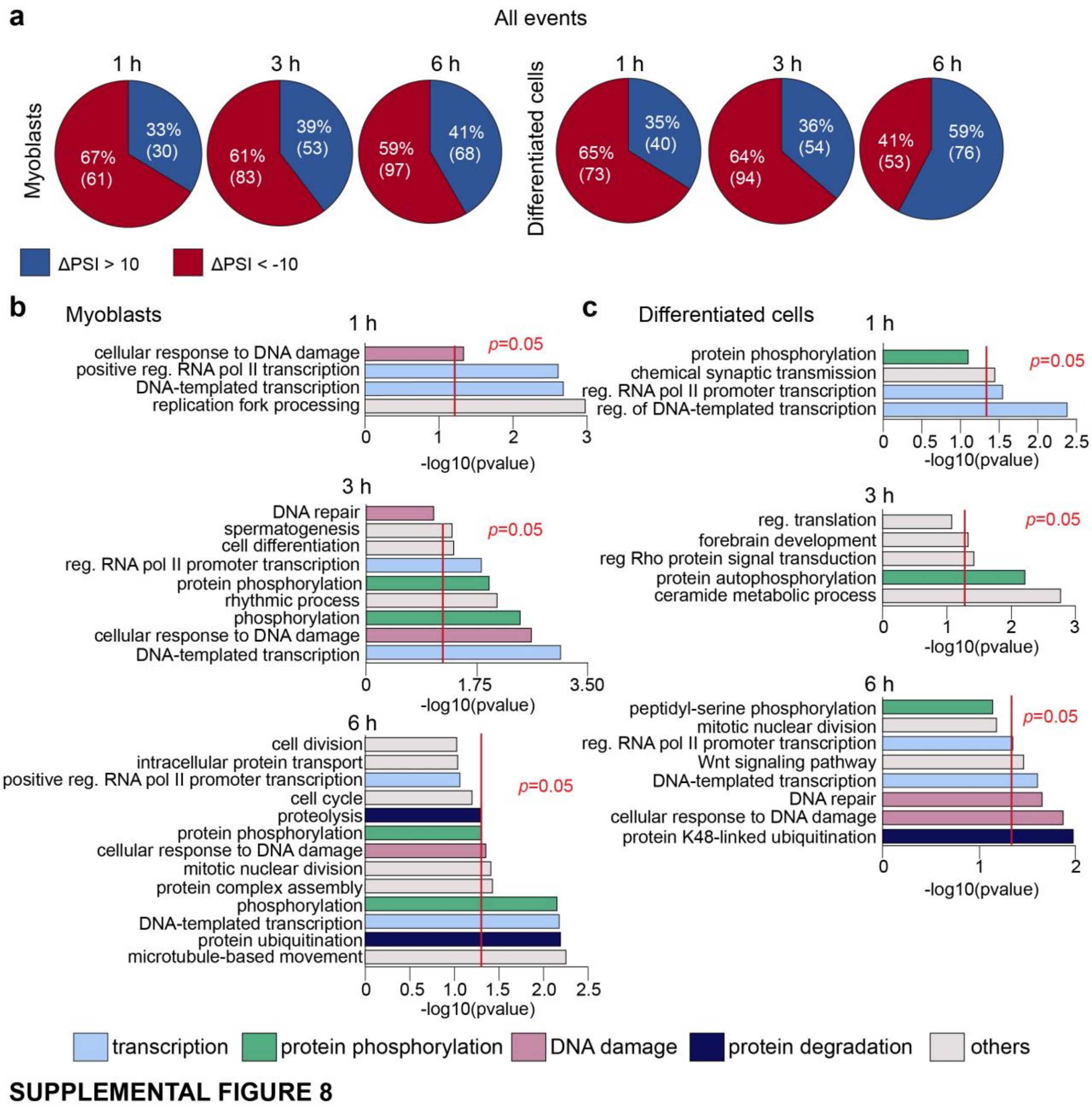

Supplemental Figure 8. Stretching induces exon inclusion in genes encoding proteins involved in transcription, DNA damage, degradation, and phosphorylation. a. Proportion of splicing events that underwent more inclusion (ΔPSI > 10, blue) or more exclusion (ΔPSI < -10, red) of the alternatively spliced region after stretching in muscle cells. The numbers between parentheses indicate the number of splicing events. The ΔPSI was defined as the difference between the PSI in stretched

samples and the PSI in the non-stretched controls. GO analysis was performed using DAVID on the cassette exons alternatively spliced after 1 h, 3 h, or 6 h stretching of myoblasts (**b**) and differentiated cells (**c**). Red line indicates *p*-values for the GO analysis of 0.05. Events were considered alternatively spliced if $|\Delta PSI| > 10$. PSI: percent spliced in. Reg: Regulation.

a

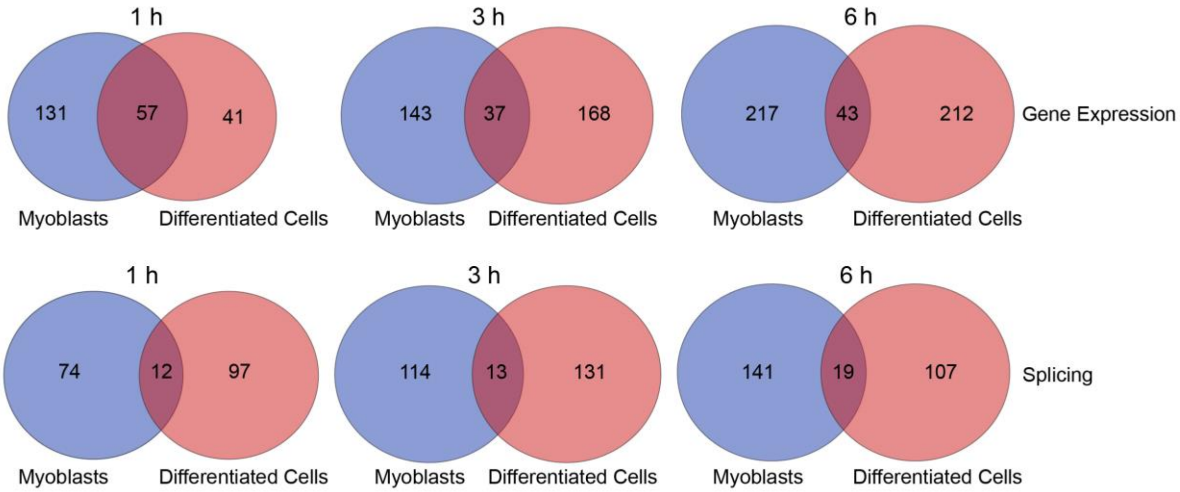

b

Gene symbol	Gene name
Fos	Fos proto-oncogene
Ctgf	Connective tissue growth factor
Cyr61	Cysteine-rich angiogenic inducer 1
Atf3	Activating transcription factor 3
Bmf	Bcl2 modifying factor
Dusp1	Dual specificity phosphatase 1
Egr1	Early growth response 1
Nr4a1	Nuclear receptor subfamily 4 group A member 1
Stat2	Signal transducer and activator of transcription 2
Sgk1	Serum/glucocorticoid regulated kinase 1
Mylip	Myosin regulatory light chain protein
Hoxa3	Homeobox A3

SUPPLEMENTAL FIGURE 9

Supplemental Figure 9. Overlap of mechanosensitive genes at the transcriptional or splicing levels between myoblasts and differentiated cells. a. The mechanosensitive genes (both at the transcriptional and splicing levels) in myoblasts and differentiated cells were overlapped. **b.** Examples of some of the mechanosensitive genes (1 h stretching). Genes labeled in green encode proteins that are involved in the MAPK pathway.

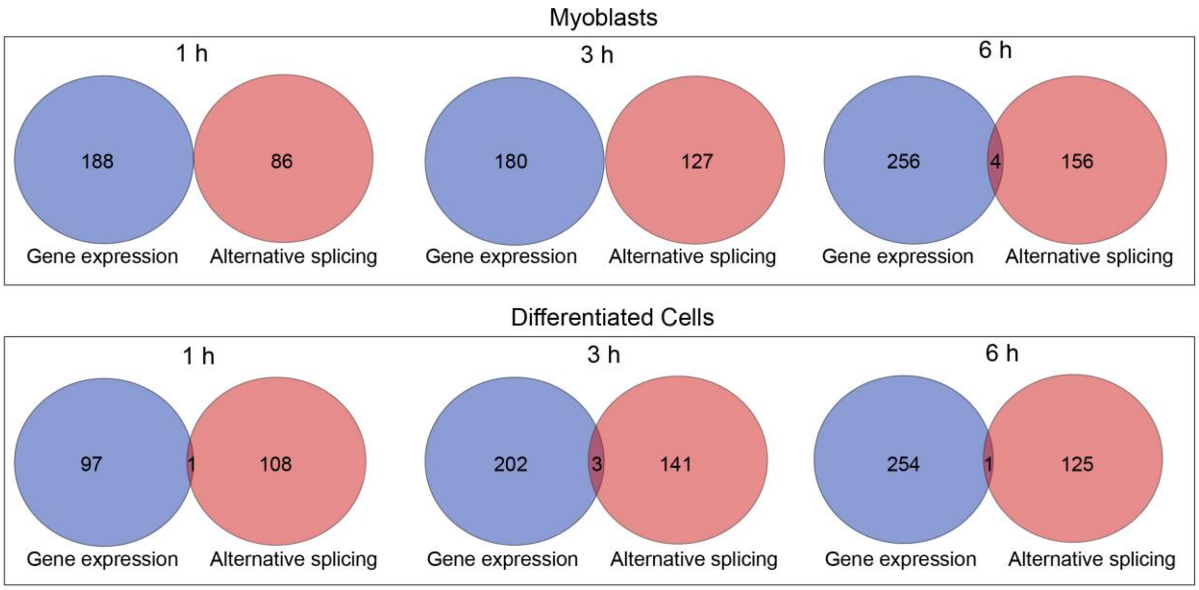

SUPPLEMENTAL FIGURE 10

Supplemental Figure 10. Genes that are mechanosensitive at the transcriptional level are distinct from those changing their splicing patterns. The mechanosensitive genes at the transcriptional level were overlapped with those changing their alternative splicing patterns in response to stretching for each time point (1 h, 3 h, 6 h) and cellular condition (myoblasts, differentiated cells).

SUPPLEMENTAL TABLES

Supplemental Table 1. Genes validated via qPCR and identity of the utilized qPCR probes which were purchased from Thermo Fisher Scientific.

Gene symbol	Probe
Acat2	Mm00782408_s1
Atf3	Mm00476033_m1
Ccl2	Mm00441242_m1
Clk1	Mm01255276_m1
Ctgf	Mm01192933_g1
Cyr61	Mm00487498_m1
Egr1	Mm00656724_m1
Ereg	Mm00514794_m1
Fos	Mm00487425_m1
Fzd6	Mm00433387_m1
Heyl	Mm00516558_m1
Hmgcs1	Mm01304569_m1
Hmox1	Mm00516005_m1
Insig1	Mm00463389_m1
Myc	Mm00487804_m1
Myog	Mm00446194_m1
Neat1	Mm01720914_g1
Nr4a1	Mm01300401_m1
Ppp1r15a	Mm01205601_g1
Rrsp1	Mm00502719_m1
Sdf2l1	Mm00452079_m1
Slc20a1	Mm00489378_m1
Srsf4	Mm00491080_m1
Srsf5	Mm00833629_g1
Srsf6	Mm00471475_m1
Txnip	Mm01265657_g1
