# Supplementary material for: Stretching muscle cells induces transcriptional and splicing transitions and changes in SR proteins
Source: Commun Biol. 2022 Sep 19;5:987. doi: 10.1038/s42003-022-03915-7 (PMC9485123; doi:10.1038/s42003-022-03915-7)
Supplement: Supplementary file 3 — Description of Additional Supplementary Files [file 42003_2022_3915_MOESM3_ESM.pdf]

Description of Additional Supplementary Files

File name: Supplemental Data 1

Description: Mapping rates of RNA-sequencing samples

File name: Supplemental Data 2

Description: Significant gene expression changes for myoblasts in excel format

File name: Supplemental Data 3

Description: Significant gene expression changes for differentiated cells in excel format

File name: Supplemental Data 4

Description: Significant alternative splicing changes for myoblasts in excel format

File name: Supplemental Data 5

Description: Significant alternative splicing changes for differentiated cells in excel format

File name: Supplemental Data 6

Description: PCR Primers for alternative splicing analysis
