# Supplementary material for: Stretching muscle cells induces transcriptional and splicing transitions and changes in SR proteins
Source: Commun Biol. 2022 Sep 19;5:987. doi: 10.1038/s42003-022-03915-7 (PMC9485123; doi:10.1038/s42003-022-03915-7)
Supplement: Supplementary file 10 — Reporting Summary [file 42003_2022_3915_MOESM10_ESM.pdf]

nature portfolio

Corresponding author(s): Jimena Giudice

Last updated by author(s): Jul 26, 2022

Reporting Summary

Nature Portfolio wishes to improve the reproducibility of the work that we publish. This form provides structure for consistency and transparency in reporting. For further information on Nature Portfolio policies, see our Editorial Policies and the Editorial Policy Checklist.

Statistics

For all statistical analyses, confirm that the following items are present in the figure legend, table legend, main text, or Methods section.

n/a	Confirmed	
☐	☒	The exact sample size (*n*) for each experimental group/condition, given as a discrete number and unit of measurement
☐	☒	A statement on whether measurements were taken from distinct samples or whether the same sample was measured repeatedly
☐	☒	The statistical test(s) used AND whether they are one- or two-sided<br>*Only common tests should be described solely by name; describe more complex techniques in the Methods section.*
☒	☐	A description of all covariates tested
☐	☒	A description of any assumptions or corrections, such as tests of normality and adjustment for multiple comparisons
☒	☐	A full description of the statistical parameters including central tendency (e.g. means) or other basic estimates (e.g. regression coefficient) AND variation (e.g. standard deviation) or associated estimates of uncertainty (e.g. confidence intervals)
☒	☐	For null hypothesis testing, the test statistic (e.g. *F*, *t*, *r*) with confidence intervals, effect sizes, degrees of freedom and *P* value noted<br>*Give P values as exact values whenever suitable.*
☒	☐	For Bayesian analysis, information on the choice of priors and Markov chain Monte Carlo settings
☒	☐	For hierarchical and complex designs, identification of the appropriate level for tests and full reporting of outcomes
☒	☐	Estimates of effect sizes (e.g. Cohen's *d*, Pearson's *r*), indicating how they were calculated

Our web collection on statistics for biologists contains articles on many of the points above.

Software and code

Policy information about availability of computer code

Data collection	We generated RNA for RNA-seq and sent it to the Bioinformatics Core who then collected the data for us.
Data analysis	For gene expression, RNA-seq samples were aligned to the Ensembl mm10 mouse genome and mm10 mouse transcriptome using STAR (2.7.6a). Expression was quantified for downstream analysis using Salmon (1.4.0). Gene expression was calculated using DESeq2 (1.30.1). For alternative splicing, RNA-seq samples were aligned to the Gencode mm10 mouse genome and the Gencode vM20 transcriptome. Differential splicing was determined using MISO (2017 version).

For manuscripts utilizing custom algorithms or software that are central to the research but not yet described in published literature, software must be made available to editors and reviewers. We strongly encourage code deposition in a community repository (e.g. GitHub). See the Nature Portfolio guidelines for submitting code & software for further information.

Data

Policy information about availability of data

All manuscripts must include a data availability statement. This statement should provide the following information, where applicable:

- Accession codes, unique identifiers, or web links for publicly available datasets
- A description of any restrictions on data availability
- For clinical datasets or third party data, please ensure that the statement adheres to our policy

The RNA-seq data generated during the current study will be available in the GEO repository under accession number GSE190029 upon article publication.

Field-specific reporting

Please select the one below that is the best fit for your research. If you are not sure, read the appropriate sections before making your selection.

☒ Life sciences ☐ Behavioural & social sciences ☐ Ecological, evolutionary & environmental sciences

For a reference copy of the document with all sections, see nature.com/documents/nr-reporting-summary-flat.pdf

Life sciences study design

All studies must disclose on these points even when the disclosure is negative.

Sample size	We performed n=4 for all our timepoints in both myoblasts and differentiated cells to have enough replicates to generate strong RNA-seq data and also to balance the cost of deep RNA-sequencing.
Data exclusions	No data was excluded from the analyses.
Replication	We successfully validated the accuracy of RNA-sequencing by verifying gene expression and alternative splicing changes. We also successfully replicated gene expression and alternative splicing changes found by RNA-seq in other samples that were stretched but not sent for RNA-seq.
Randomization	Randomization was not relevant to our study because we wanted to determine the effect of well-controlled cell stretching on gene expression and alternative splicing.
Blinding	The investigators were not blinded to elements of the study because it is clear during the experiment which cells are being stretched and which cells are not being stretched. Blinding is also unnecessary because there is no subjectivity in these experiments and the results are quantitative.

Reporting for specific materials, systems and methods

We require information from authors about some types of materials, experimental systems and methods used in many studies. Here, indicate whether each material, system or method listed is relevant to your study. If you are not sure if a list item applies to your research, read the appropriate section before selecting a response.

Materials & experimental systems

n/a	Involved in the study	
☐	☒	Antibodies
☐	☒	Eukaryotic cell lines
☒	☐	Palaeontology and archaeology
☒	☐	Animals and other organisms
☒	☐	Human research participants
☒	☐	Clinical data
☒	☐	Dual use research of concern

Methods

n/a	Involved in the study	
☒	☐	ChIP-seq
☒	☐	Flow cytometry
☒	☐	MRI-based neuroimaging

Antibodies

Antibodies used	Anti-phosphoERK1/2 from Cell Signaling (#4370, 1:2,000), anti-ERK1/2 from Cell Signaling (#4695, 1:2,000), anti-SRSF4 from Millipore Sigma (#06-1367, 1:500), anti-SRSF5 from Millipore Sigma (#06-1365, 1:500), anti-SRSF6 from Bethyl Laboratories (A303-669A-T, 1:1,000), and anti-phosphorylated SR proteins from Sigma Aldrich (MABE-50, 1:750)
Validation	We performed siRNA knockdown experiments of SRSF4, SRSF5, and SRSF6 and validated that the SR protein antibodies were identifying the correct band and that it was the same molecular weight as the band identified using the phospho-SR protein antibody. The phosphoERK1/2 and ERK 1/2 antibody is widely used and has been published in 5,909 and 4,447 publications respectively.

Eukaryotic cell lines

Policy information about cell lines

Cell line source(s)	C2C12 cells were purchased from ATCC (CRL-1722)
Authentication	We regularly authenticate the C2C12 cells in the lab in in several ways. We profile known myogenic markers in myoblasts and differentiated cells (myotubes) by real time PCR (qPCR) . We also phenotypically characterize cellular differentiation through immunofluorescence by staining a muscle myosin heavy chain and calculating the level of fusion (fusion index). Finally, we check various markers in both myoblasts and differentiated cells by western blot assays.

Mycoplasma contamination	Prior to us purchasing the cells they were verified to not be contaminated with mycoplasma. We regularly stain our cells with DAPI and we didn't detect any punctate pattern staining typical of mycoplasma contamination. We also tested our cells for mycoplasma using a PCR kit and did not detect any mycoplasma.
Commonly misidentified lines (See ICLAC register)	N/A
